# Targeting choroid plexus epithelia and ventricular ependyma for drug delivery to the central nervous system

**DOI:** 10.1186/1471-2202-12-4

**Published:** 2011-01-07

**Authors:** Ana Maria Gonzalez, Wendy E Leadbeater, Michael Burg, Karen Sims, Tetsuya Terasaki, Conrad E Johanson, Edward G Stopa, Brian P Eliceiri, Andrew Baird

**Affiliations:** 1 School of Experimental Medicine and Dentistry, University of Birmingham, Edgbaston, UK; 2 Division of Trauma, Burns and Surgical Critical Care, Department of Surgery, University of California San Diego, San Diego Calif. USA; 3 School of Pharmaceutical Sciences, Tohoku University, Japan; 4 Neurosurgery and Neuropathology, Rhode Island Hospital of Brown University, Providence RI USA

## Abstract

**Background:**

Because the choroid plexus (CP) is uniquely suited to control the composition of cerebrospinal fluid (CSF), there may be therapeutic benefits to increasing the levels of biologically active proteins in CSF to modulate central nervous system (CNS) functions. To this end, we sought to identify peptides capable of ligand-mediated targeting to CP epithelial cells reasoning that they could be exploited to deliver drugs, biotherapeutics and genes to the CNS.

**Methods:**

A peptide library displayed on M13 bacteriophage was screened for ligands capable of internalizing into CP epithelial cells by incubating phage with CP explants for 2 hours at 37C and recovering particles with targeting capacity.

**Results:**

Three peptides, identified after four rounds of screening, were analyzed for specific and dose dependant binding and internalization. Binding was deemed specific because internalization was prevented by co-incubation with cognate synthetic peptides. Furthermore, after i.c.v. injection into rat brains, each peptide was found to target phage to epithelial cells in CP and to ependyma lining the ventricles.

**Conclusion:**

These data demonstrate that ligand-mediated targeting can be used as a strategy for drug delivery to the central nervous system and opens the possibility of using the choroid plexus as a portal of entry into the brain.

## Background

Of the many strategies to defeat and bypass the blood brain barrier (BBB) for the purposes of drug delivery [1,2], the unique biological and biophysical features of the choroid plexus (CP), its intrinsic ability to produce cerebrospinal fluid (CSF) and its capacity to place bioactive proteins into CSF [3-11] have largely been ignored. More recently, there are a number of reports suggesting the possibility that secretory epithelial cells of the CP present a potential window into the central nervous system (CNS) for the purposes of drug delivery [5,9,12-14]. On one hand, if CP-epithelial drug targeting strategies could be developed and validated, then drugs, proteins and gene based medicines might be delivered to the CP for their translocation into CSF and availability to brain parenchyma. Alternatively, drug-targeting to the CP epithelial cells could be used to alter unique functions of CP epithelial cells that include CSF production and the transport in, and out, of the CNS of critical components like ions, vitamins, hormones and proteins like ascorbic acid, amyloid beta protein [5].

In as much as CSF-producing CP epithelia are distinct cellular structures within the CNS and anatomically accessible via a fenestrated endothelium, we wondered whether phage display might be used to identify targeting ligands capable of delivering drugs into CP epithelial cells. As a first step towards this goal, our previous studies identified epidermal growth factor (EGF) as a potential ligand capable of mediating particle internalization into target cells [15-19] and we showed its ability to enter CP epithelia in vitro and transduce them *in vivo *[20]. Like the receptors for adenoviral and lentiviral gene delivery vectors which can also transduce choroid epithelial cells after i.c.v. injection [21,22], the EGF receptor is widely distributed. Moreover, the ligand, EGF, has intrinsic epidermal cell growth promoting biological activity [23,24] that significantly limits its utility as a drug- and particle-targeting agent.

In this study, we attempted to validate an approach that might increase CP targeting specificity. To this end, we show that a combinatorial peptide library containing over 10^9 ^different peptides can be selectively mined for ligands that allow binding and internalization into mouse CP explants. Three ligand-targeting phage recovered by biopanning have the capacity to target and internalize particles into CP epithelial cells in culture using TCSFB cells. Furthermore, after i.c.v. injection into lateral ventricles, the targeted phage internalize into CP epithelial cells and ependyma cells. Because the targeted binding is specific and dose dependent, the possibility arises that these targeted phage may be candidates to re-engineer CNS gene delivery vectors and drug targeting to the CNS.

## Results

### Biopanning PhD-C7C libraries of phage for peptides internalizing into CP Explants

As shown in Table 1, three recovered peptides named "Peptide B" (R3P3.9), "Peptide C" (R4P4.5) and "Peptide D" (R4P4.7) were selected from the 25 unique sequences obtained for further analysis from 3-4 rounds of biopanning on explants of 4^th ^ventricle mouse choroid plexus. A 5^th ^round of biopanning did not provide further enrichment of library collapse and was not studied. Freshly dissected choroid plexus were incubated with the 1 × 10^11 ^phage for 2 hours as indicated in Materials and Methods, unbound phage washed off and any phage particles that had not washed off were presumed to be internalized and were recovered by phage display biopanning [16,25]. No sequencing was performed after the first and second rounds of biopanning but of the 19 sequences obtained after Round 3, the data suggested that the original library had collapsed to 5 families of sequences of which 3 peptides (R3P3.9, R3P3.23 and R3P3.24) were identical. Representing the Family 1 of sequences, the candidate (CHGNHMPAC) was called "Peptide B". On the 4^th ^round of screening, this peptide B sequence (R3P3.9) was not recovered but a peptide identified in Round 3 (R3P18) and representing Family 4 sequences like R3P18, R3P6, R3P3.12 and R3P3.10 detected in Round 3 was identified in Round 4 (R4P15). As such this sequence (CSASTHKAC) was selected for further evaluation and called "Peptide C". A 2^nd ^identical member of this 4^th ^family was detected in both Rounds 3 (R3P3.6) and 4 (R4P4.7) and this peptide (CTSTSYKMC) was called Peptide D. Wild type phage with no peptide insert was called "Peptide A" and each particle tested blind for binding and internalization.

**Table 1:** Identification of CP-targeting peptides.

Clone	Sequence	Family	Repeat	Clone	Sequence	Family	Repeat
3.1	CHERPSSLC	3		4.2	CKALDATTC	4	

3.2	CPAVVRGSC	5		4.5	CSASTHKAC	4	

3.3	CDHRSSAQC	3		4.7	CTSTSYKMC	5	3.6/4.15

3.5	CLRSQGPVC	1		4.8	CTKMHLPQC	6	

3.6	CTSTSYKMC	4	4.7	4.11	CAPHSTSAC	5	

3.7	CQPTHSQSC	2		4.12	CHHPQNRHC	6	

3.8	CSMPHLTSC	5		4.15	CSASTHKAC	4	3.18/4.5

3.9	CHGNHMPAC	1	3.23/3.24	4.16	CHGPIDKSC	6	

3.10	CKSIPTNAC	4		4.19	CIPTSIRHC	5	

3.12	CMSGKHLKC	4		4.23	CSPKGSHSC	5	

3.15	CLRDDSHSC	1					

3.16	CHHQHSPQC	1					

3.17	CEWPTSSTC	3					

3.18	CSASTHKAC	4	4.15				

3.19	CNSHASPHC	1					

3.21	CIIGQGPSC	1					

3.22	CMMPSRSLC	5					

3.23	CHGNHMPAC	1	3.24/3.9				

3.24	CHGNHMPAC	1	3.23/3.9				

Peptide sequences obtained from after 3 and 4 rounds of biopanning were compared, assigned to one of six different families of sequences and repeat recoveries noted as described in the text.

### Targeted peptide phage are internalized by cells in CP explants

**Figure 1:**
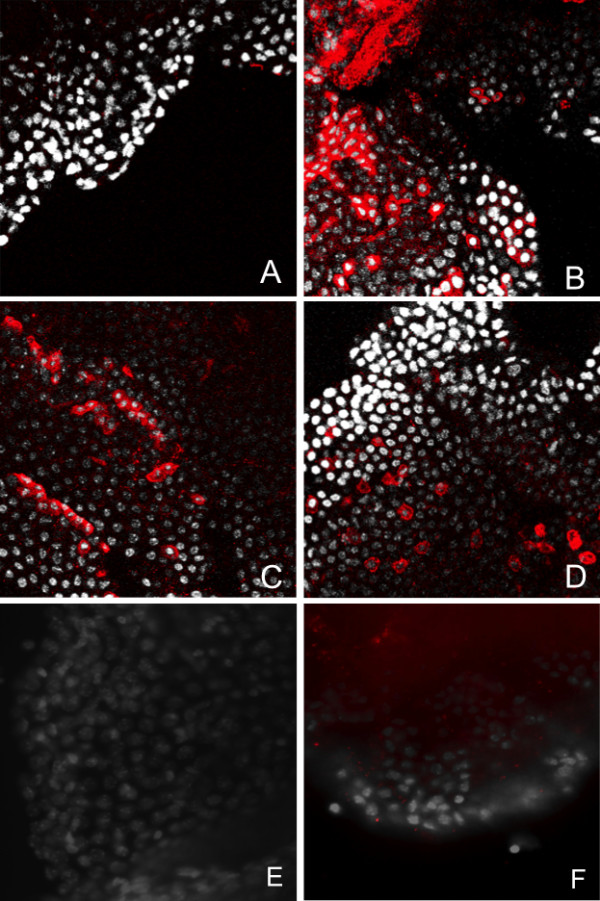
**Targeted-phage internalize into cells in CP explants in a ligand dependant fashion**. Choroid plexus was dissected from the lateral and 4^th ^ventricle and incubated with PhD-targeted phage (1 × 10^12^pfu/ml) for 2 h at 37C. To remove any particles bound to the cell surface, the explants were washed with PBS containing Tween 20, fixed and then immunostained with antibodies against M13 phage. Binding of primary antibodies was detected using Alexa^594^-goat anti rabbit antibodies. No immunostaining was observed when choroid plexus explants were incubated with Peptide A control phage (Panel A). In contrast, immunoreactive M13 staining was observed in the cytoplasm of selected populations of epithelial cells after incubation with Peptide B (Panel B), Peptide C (Panel C) or Peptide D (Panel D) targeted phage indicating the internalization of these targeted particles by epithelial cells in the choroid plexus. To demonstrate that internalization is ligand-mediated, explants were co-incubated with 200 ng/ml Peptide C (Panel E) or Peptide D (Panel F) and the targeted phage. M13 phage: red; Cell nuclei (DAPI): white.

We used immunohistochemistry to evaluate whether the particles identified by biopanning internalized into CP explants and behaved as predicted (Figure 1). Mouse CP were dissected from either the lateral or 4^th ^ventricle as described in Material and Methods and incubated with the untargeted phage A or the peptide targeted B-, C- or D-particles at a concentration of 1 × 10^11^pfu/ml for 2 h at 37C. To remove any particles that might be non specifically bound to the cell surface, the explants were washed with PBS containing Tween-20, fixed in methanol and then immuno-stained with antibodies against M13 phage as described in Materials and Methods. Binding of primary antibodies was detected using Alexa^594^-goat anti-rabbit antibodies. As shown in Figure 1, little immunostaining is observed when choroid plexus explants are incubated with Peptide-A (untargeted) control phage (Panel A). In contrast, immunoreactive M13 staining is observed in the cytoplasm of what appears to be selected populations of epithelial cells after incubation with particles targeted with Peptide-B (Panel B), Peptide-C (Panel C) or Peptide-D (Panel D) targeted phage indicating the internalization of these targeted particles by epithelial cells in the choroid plexus. We noted regional differences in the internalization and distribution of targeted cells in the explants with each targeted phage. While we consistently observed non-uniform internalization, it was not possible to determine whether this pattern represented restricted availability of particles to the explants or the existence of unique cell specific distributions of targeted receptors.

### Peptide-targeting is specific

As also shown in Figure 1, CP explants were treated with either media (Panel A), 200 ng/ml of synthetic Peptide C (Panel E) or 200 ng/ml of Peptide-D (Panel F) in addition to either 1 × 10^11^pfu/ml of Peptide-C targeted phage (Panels A&C) or 1 × 10^11^pfu/ml of Peptide-D targeted phage (Panels B&D) for 2 h at 37C. Prior to staining, cells were washed with 50 mM Glycine, pH 2.8, fixed and immunostained with antibodies against M13 phage. Binding of primary antibodies was detected using Alexa^594^-goat anti rabbit antibodies. After 2 h, intense intracellular immunoreactivity was observed in cells incubated with PhD C targeted phage (Panel C) or Peptide-D targeted phage (Panel D). When explants were pretreated with either Peptide-C (Panel E) or Peptide-D (Panel F), no specific staining was observed in the explants indicating that the internalization of the Peptide-C and Peptide-D targeted phage was displaced by the excess peptide.

### Internalization of PhD-targeted peptide phage in CP cells in vitro

**Figure 2:**
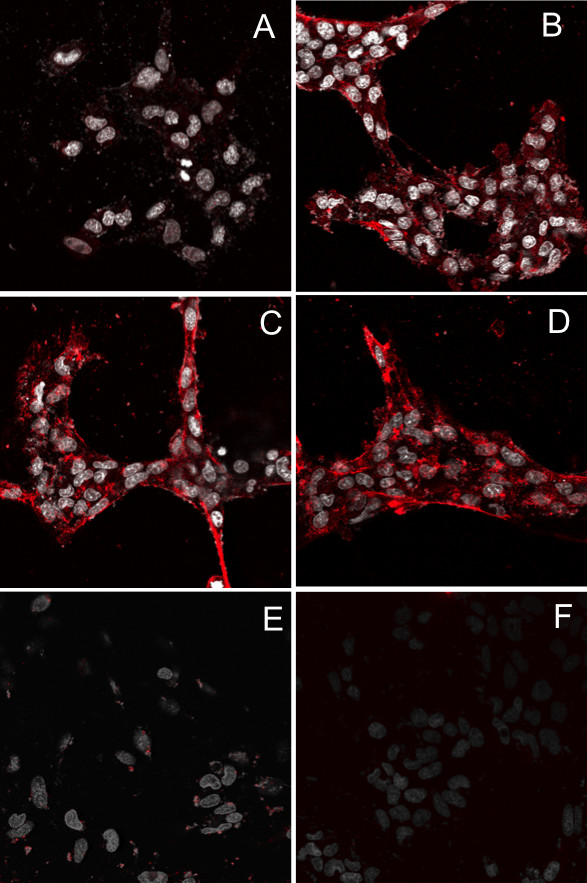
**Internalization and specificity of PhD-targeted peptide phage in CP cells in culture**. TR-CSFB cells were incubated with untargeted or targeted phage (5 × 10^10^pfu/ml) for 2 h at 37C, acid washed to remove any particles on the cell surface, fixed and then immunostained with antibodies against M13 phage and subsequently Alexa^594^-labeled goat anti rabbit antibodies. Little internalization is observed when the cells were treated with control phage (Panel A) and while cells tended to clump together upon staining, significant intracellular immunoreactivity was nevertheless observed in TR-CSFB cells after incubation with Peptide B (Panel B), Peptide C (Panel C) or Peptide D (Panel D) targeting of phage. M13 phage: red; Cell nuclei (DAPI): white. To demonstrate that internalization was ligand-mediated, TR-CSFB cells were incubated with targeted phage (1 × 10^10^pfu/ml) for 2 h at 37C in the absence (Panel A) or presence of an excess (200 ng) of Peptide C (Panel E) or Peptide D (Panel F). M13 phage: red; Cell nuclei (DAPI): white.

To confirm epithelial cell targeting and to determine whether there existed specificity for epithelial cell subpopulations, rat choroid plexus epithelial cells from the TR-CSFB cell line were incubated with Peptide-A (control), Peptide-B, Peptide-C or Peptide-D targeted phage (5 × 10^10^pfu/ml) for 2 h at 37C, then acid washed to remove any particles on the cell surface, fixed and immunostained with antibodies against M13 phage (Figure 2). Binding of primary antibodies was detected using Alexa^594^-goat anti rabbit antibodies. No internalization is observed when the cells are treated with control PhD A phage (Panel A). Significant intracellular immunoreactivity is observed in TR-CSFB cells after incubation with PhD B (Panel B), PhD C (Panel C) or PhD D (Panel D). M13 phage, red; Cell nuclei (DAPI), white. The TR-CSFB cells were also co-incubated with excess of free Peptide-C (Panel E) or Peptide-D (Panel F) and cells were washed with 50 mM Glycine, pH 2.8, fixed and then immunostained with antibodies against M13 phage. In both instances, internalization is almost completely blocked when the targeted particles are competing for binding to cell surface receptors with an excess of ligand.

### Internalization of PhD targeted-phage is dose and time dependant

**Figure 3:**
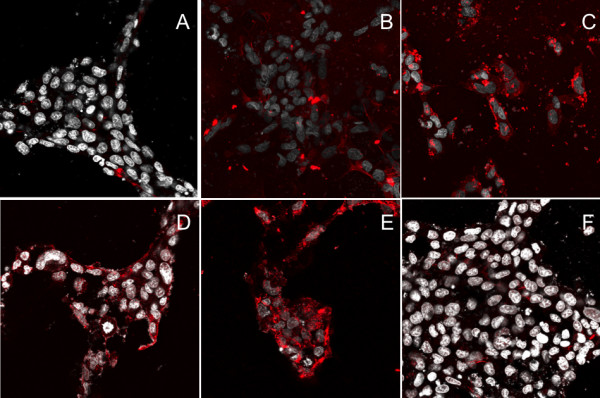
**Internalization of PhD targeted-phage is dose dependant in CP cells in vitro**. TR-CSFB cells were incubated with increasing concentrations of Peptide D-targeted phage for 2 h, at 37C. Cells were acid washed to remove cell surface binding, immunostained with antibodies against M13 phage and subsequently detected using Alexa^594^-labeled goat anti-rabbit antibodies. At 1 × 10^9 ^pfu/ml, no significant intracellular staining was observed (Panel A). At 1 × 10^10 ^pfu/ml (Panel B), there was detectable immunoreactivity for M13 in the cytoplasm which clearly increased when the cells were incubated with 1 × 10^11 ^pfu/ml (Panel C). This experiment shows that there was a correlation between phage titer and the intensity of intracellular immunoreactivity indicating that internalization is dose dependant. Internalization of targeted-phage was also time-dependant (Panels D-E). TR-CSFB cells were incubated with Peptide D-targeted phage (5 × 10^10^pfu/ml) at 37C, acid washed, immunostained with M13 antibodies and internalization visualized using Alexa^594 ^labeled secondary antibodies. Cells were counterstained with DAPI to visualize the cell nuclei. By 15 min, Peptide D-targeted phage were already internalized (Panel D) and this intracellular staining increases over the following 2 h (Panel E) but particle is degraded by 48 h (Panel F). M13 phage: Red; Cell nuclei: white.

To evaluate the binding kinetics of phage internalization, TR-CSFB cells were incubated with increasing concentrations of Peptide-D-targeted phage for 2 h, at 37C (Figure 3). Cells were acid washed to remove cell surface binding, immunostained with antibodies against M13 phage and detected using Alexa^594 ^goat labeled anti-rabbit antibodies. At 1 × 10^9 ^pfu/ml, little significant intracellular staining is observed (Panel A) but at 1 × 10^10 ^pfu/ml (Panel B) there is detectable immunoreactivity for M13 in the cytoplasm which clearly increases when the cells are incubated with 1 × 10^11 ^pfu/ml (Panel C). This experiment shows that there is a clear dose dependant correlation between phage titer and intensity of intracellular immunoreactivity. Similarly, if TR-CSFB cells were incubated with Peptide-D targeted phage (5 × 10^10^pfu/ml) and acid washed, immunostained and visualized analyzed at different times, by 15 min, Peptide-D-targeted phage are already internalized (Panel D) and the intracellular staining increases by 2 h (Panel E). By 48 h (Panel F), there is a reduction on the amount of immunoreactive M13 which by 72 his negligible non detectable (not shown).

### Targeted phage are internalized by choroid epithelial cells and ventricular epithelial cells after ICV injection

**Figure 4:**
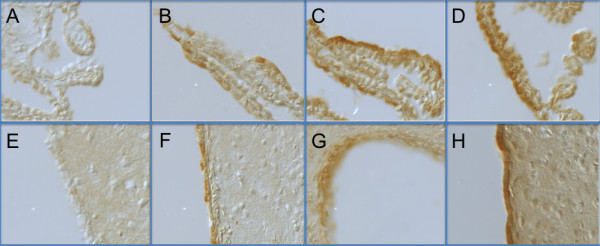
**Peptide-targeted phage are internalized by choroid epithelial cells and ependyma 24 h after icv injection**. Control phage (Panel A), Peptide B (Panel B), Peptide C (Panel C) or Peptide D (Panel D) -targeted phage (1 × 10^11^/10 ul) were injected into the lateral ventricle of anesthetized rats and 24 h later brain were collected and frozen sections immunostained with anti-M13 antibodies. Whereas low background immunoreactivity was observed in choroid plexus epithelial cells after control (untargeted) phage injections (Panel A), Peptide B (Panel B), Peptide C (Panel C) and Peptide D (Panel D) -targeted phage injections resulted in strong M13 immunoreactivity was observed within most of the epithelial cells of the choroid plexus. Similarly, very low immunoreactivity was observed in the ependymal cells after a wild type phage injection (Panel E) but after Peptide B (Panel F), Peptide C (Panel G) and Peptide D (Panel H) targeting, ependymal cells of the lateral ventricle clearly show intracellular immunostaining. Notice the lack of M13 immunoreactivity observed in the brain parenchyma in all treatments.

In Figure 4, control Peptide-A (Panel A) or targeted Peptide-B (Panel B), Peptide-C (Panel C) or Peptide-D (Panel D) phage were injected into the lateral ventricle of rats and 24 h later brains were collected and 10 um sections immuno stained with anti-M13 antibodies. Very low immunoreactivity was observed in the choroid plexus after wild type phage injection (Panel A). However, after P Peptide-B (Panel B), Peptide-C (Panel C) or Peptide-D (Panels D) targeted phage injections, strong M13 immunoreactivity was observed within most of the epithelial cells of the choroid plexus. Little staining is detected in brain parenchyma although non-specific targeting was detected at the level of the arachnoid membranes (not shown). Very low immunoreactivity was observed in the ependymal cells after control phage injections (Panel E) but after Peptide-B targeted phage injections selected populations of ependymal cells were strongly stained for M13. (Panel F). After Peptide-C targeted and Peptide-D targeted phage i.c.v. injections, ependymal cells of the lateral ventricle clearly show intracellular and uniform immunostaining (Panel G and Panel H).

## Discussion

We selected three peptides to show that it is possible to identify drug-targeting candidates for CNS drug delivery from a phage peptide library. Our biopanning strategy for targeting generated over two dozen new sequences that we predicted would be biased for luminal targeting of CP epithelial cells because the screen used CP explants freshly dissected from donor mice. The three peptides were selected because they were found to have either increased representation in the limited number of clones sequenced (e.g. R3.9) or alternatively whose sequences appeared after 3^rd ^and 4^th ^rounds of biopanning. These peptides, along with EGF [20], are proven capable of targeting CP epithelial cells in vitro, ex vivo and in vivo. Whether they can target cells for transduction after i.c.v. injection like EGF [20] remains to be established.

In 1985, Smith [26] conceived of phage display as a method whereby short nucleic acid sequences of DNA could be inserted into the coding sequence of the M13 phage glll gene to generate particles that display on their capsid surface a peptide-plll fusion protein. Reasoning that the displayed peptides would confer phage with new intrinsic activities, he proposed that it should be possible to introduce random and known sequences of DNA into the glll gene to create particles with new activities. Over the last twenty years, we and other investigators have been adapting this original phage display technique to identify novel peptides with different specificities and activities (reviewed in [27,28]), for example, peptides have that confer physical stability to particles in organic solvents like chloroform, decrease complement activation of macromolecules in blood, modify immunogenicity, alter viral tropism *in vitro *and *in vivo*, internalize particles and transduce cells, promote transcytosis *in vitro *and *in vivo *and even promote transmigration of particles across cell barriers *in vitro *and *in vivo *[29-53].

Our laboratories' focus has been to identify and exploit ligands that internalize into target cells with a long-term objective of improving the specificity of drug, protein and gene-based medicine delivery. To this end, we have re-engineered phage vectors for increased binding to mammalian cells and monitored their entry into cells by immunohistochemistry for internalization drug delivery, transfection for nucleotide delivery and transduction for gene expression [15-19,25,54-58]. It seemed reasonable to propose that these methods might deployed towards CNS drug delivery because ligands like EGF can target the phage particle to cells [20].

Several years ago, investigators [14] proposed that it might be possible to target therapeutics to the CNS by exploiting one of the unique features of the choroid plexus: it exists at the interface between blood and CSF. If possible, drugs could then be designed to act like ascorbic acid and translocate across the CP epithelium for entry into CSF and access to brain parenchyma [59-61]. Alternatively, drugs could be targeted to the CP epithelium so as to modify its hydrodynamic and homeostatic functions in controlling CSF production and composition respectively. We show here that when phage are re-engineered so that they can target the choroid plexus epithelium, they internalize into epithelial cells when tested in vitro, ex vivo and in vivo. The targeting is ligand specific, concentration and time dependant.

The possibility of exploiting epithelial routes of drug delivery to the CNS is not new. For example, we originally proved the concept of CP targeting with the ligand EGF [20] but it was clear from the outset that this ligand is not ideally suited for CP targeting. First, EGF has intrinsic activity and is a naturally occurring growth factor. Second, while EGF may be selective for epithelial cells in the brain as described here, but there are several alternative cell types that express EGF receptors including astrocytes, activated glia and endothelial cells. The specificity that we observe with i.c.v. injections is likely due to the compartmentalization of phage in the ventricular space and the limited number of cell types available for targeting. Herenu et [62] also explored the ependymal route for insulin like growth factor-1 (IGF-1) gene delivery in an attempt to circumvent the need to transport IGF-1 from blood into brain by injecting peptide i.c.v. [63,64]. In this case, they exploited adenoviral selectivity to infect epithelial cells when injected i.c.v. to increase the levels of IGF1 in CSF and mimic the concentrations achieved by the injection of protein into CSF. Presumably, adenoviral-retargeting strategies that modify its pharmacokinetics, biological activity and even potency could be used to improve its use as a gene delivery agent to the CNS.

In the study described here, we have not studied the mechanism of internalization or the specific trafficking of the particles once they enter the cell. The fact that the peptide ligand can prevent internalization suggests specificity for cell binding. The observation that the particles are internalized in a ligand dependant fashion supports the hypothesis that phage bind to cells via cognate receptors to the peptides displayed. The identity of these receptors remains unknown but clearly phage display should be considered as a viable method to identify novel portals of entry into the CNS via delivery to CPe cells.

Using the CP as a doorway into the CNS has also gained momentum with significant difficulties in overcoming the blood brain barrier [1,65,66]. Just as there are reports that propose using brain's endothelium as a target for CNS drug delivery using molecular signatures[44,46,48] in brain endothelia, Chen et al [67]showed that CNS-directed enzyme therapy was feasible by targeting epitope-modified adeno-associated virus. While the effects are certainly local, the wide distribution of endothelium in the CNS would ensure wide distribution of drug throughout the brain, if all endothelia were targeted. For similar reasons, there is considerable interest in targeting the choroid epithelium [5,59]. First, the design and structure of barriers are not homogenous throughout the brain and areas like the arcuate nucleus are open to CSF while others, like median eminence, are open to the portal blood [68]. Second, the choroid plexus is actively modulating CSF content and composition in response to the environment [69] which regulate brain state and neuroactive peptide distribution in CSF[10]. Third, the choroid plexus can play a prominent role in neuroprotection [9] because of special axonal endings that are formed at CSF contacting neurons [70-72] which presumably enable communication between brain parenchymal cells and CSF. Finally, ependymal cells, which divide asymmetrically and transfer progeny into the subventricular zone activated by injury [73] are in direct contact with CSF and CP produced neuroactive agents in CSF. In as much as this subependymal zone is a neurogenic niche [74] that is influenced by CP epithelial cells and an ependymal layer controlling of CSF hydrodynamics and content, these cells are nearly ideally localized to modulate CNS functions and treat brain disease if they can be targeted to produce therapeutics. As demonstrated by Regev et al[75], lentiviral transduction of choroid plexus epithelium with genes encoding neuroactive peptides can control CNS function thereby supporting the feasibility of CP-mediated drug delivery.

## Conclusions

It is interesting to speculate that while our results support the feasibility of targeted delivery to choroid plexus epithelia and ependyma after i.c.v. injection of re-engineered particles, a more innovative therapeutic deployment would ideally involve exploiting epitopes on the basolateral epithelium that would allow targeting of particles from blood, through stroma and to the CP epithelial barrier. To this end, in vivo biopanning of basolateral CP epithelia at their stromal-endothelial interface using phage display libraries could be developed using homing techniques originally developed for vascular targeting described above [46]. Inasmuch as these approaches have already proven useful for targeting brain endothelia to modify their function [67], a similar strategy could be used to identify peptides capable of homing particles for drug transport to CP and translocation into CSF.

## Methods

### Peptide library and biopanning

All of the experiments described here used the constrained PHD-C7C library from New England Biolabs (Ipswitch, MA) that consisted of a disulfide-constrained heptapeptide (PHD-C7C). The randomized peptide segment is flanked by a pair of cysteine residues, which results in the display peptide loops rather than linear sequences. All of the libraries have reported complexities in excess of 2 × 10^9 ^independent clones and are displayed at the N-terminus of the minor coat protein pIII on M13 phage at a valency of 5 peptides per virion. The first randomized position in the PHDC7C library is preceded by Ala-Cys and all of the libraries contain a short linker sequence (Gly-Gly-Gly-Ser) between the displayed peptide and pIII. For screening libraries, we deployed a binding and internalization strategy to identify peptides capable of interacting with mouse CP explants that were dissected as described below. Tissue was incubated with libraries in DMEM culture media supplemented with 10% calf serum in tissue culture plates for 2 hours at 37C in an atmosphere of 95% CO_2 _to allow for binding and internalization. At the end of the incubation period, the explants were carefully removed from the incubation media, placed into eppendorf tubes containing extraction-lysis buffer and processed according to the manufacturers (New England Biolabs) recommendations. Phage were recovered, amplified by infection of host bacteria and, after purification by PEG precipitation, the selection repeated over four more rounds until the library had collapsed and families of related peptide sequences could be identified. For all testing on cells, endotoxin was removed byTritonX-114 phase separation[76] to eliminate non-specific internalization that accompanies LPS contamination (unpublished observations).

### Preparation of explants of mouse and rat choroid plexus

All of the experiments were performed after protocol review and approval by local Institutional Animal Care Committees as indicated below. Although the biopanning protocols used CP from Balb-C mice, we found that the 3 peptides selected for testing were effective on mouse and rat CP epithelial cell explants, regardless of the originating strain, sex and age. Accordingly, adult wild type donor C/57Bl and Balb-C mice, or alternatively Wistar and Sprague Dawley rats were used as indicated as the source of CP tissue and brains were immediately dissected and placed onto wet ice. The CP were harvested from 4^th ^ventricles by carefully separating the cerebellum from the brain stem and dissecting the choroid plexus from the roof of the cerebellum with tweezers. The lateral ventricle CP were dissected after removal of the 4^th ^ventricle's CP by immersing the brain in PBS and making two parallel sagittal incisions 10 mm from the midline along the length of the brain to a depth of 4 mm so as to cut through the corpus callosum. The cortex was then pulled away to the side exposing the lateral ventricles and choroid plexus. With a pair of tweezers, each end of the CP was gently pulled away and placed in RPMI media containing 10% fetal calf serum (FCS) and 5% normal horse serum (NHS). Experiments with CP explants were performed immediately after harvest.

### Culture of primary rat choroid plexus (CP) epithelial cells

We evaluated the effects of ligand targeting in vitro using an immortalized choroid plexus cell line (TRCSFB cells) developed from transgenic Wistar rats [77]. TRCSFB cells were plated on flasks coated with collagen type I (BD Biosciences) and cultured in DMEM + GlutaMAX (Invitrogen) supplemented with 4.5 g/L glucose, 5 ml Sodium Pyruvate (100 mM, Invitrogen), 5 ml NEAA (non-essential amino acids, ×100, (Invitrogen), 5 ml Penicillin/Streptomycin (10,000 units/ml Penicillin, 10,000 μg/ml Streptomycin, Invitrogen), and 10% FBS (Sigma).

### Incubation with phage in vitro, ex vivo and in vivo

As indicated in each of the experiments, ligand-targeted and untargeted phage particles were added to either TRCSFB cells or to the CP explants and incubated at 37C in 95% CO_2 _controlled atmosphere. Experiments with CP explants were not extended beyond 2 hours to ensure patency of the epithelial cells. At the end of the indicated incubations, the cells or tissue were rinsed first in 10 mM PBS containing Ca+2 and Mg+2, and then washed briefly in either 50 mM Glycine buffer pH 2.8 (cells in culture) or 10 mM PBS Ph 7.4 containing 0.3% Tween 20. The amount of phage bound and internalized was evaluated by immunohistochemistry.

To evaluate targeting *in vivo*, peptide-targeted and untargeted phage were injected icv into mice and rat brains as specifically indicated. All procedures involving the use of rats followed strict adherence to Home Office guidelines (UK), and with approval of the local ethics committee while all mice i.c.v. injections were approved by the Institutional Animal Care and Use Committee at the University of California San Diego. Thirty minutes prior to receiving surgery, adult male Wistar (Charles River) rats (200-250 g) received analgesia through a subcutaneous injection of 0.03 mg/kg buprenorphine. The rats were then anaesthetised using 5% isofluorane, delivered with O_2 _at 1.7 L/min through a mask, and full anaesthesia was maintained throughout the surgery.

During surgery, rats was immobilised in a stereotactic frame with the head elevated and parallel to the table surface. A sagittal incision was made along the midline of the scalp and teased apart to expose the skull. The landmark at co-ordinates 0/0 that indicate Bregma crossing the midline was marked using a fine marker pen. A small hole was drilled in the skull using a micro drill, 1.5 mm lateral to the midline, 1 mm posterior to Bregma. The needle of the Hamilton syringe was inserted 4 mm deep from the brain surface and 20 μl of phage solution was injected over 30 seconds. The syringe was left in the brain for one minute to prevent fluid reflux. Once the i.c.v. injection was complete the syringe was removed and the skin sutured, and the animal was taken off the anaesthetic and allowed to recover on a warm fleece bed. At specific times after injection (24-72 hours), the rats were humanely killed by delivering rising concentrations of CO_2 _and immediately perfused with 4% paraformaldehyde in PBS, pH 7.4. The brains were dissected and post-fixed for 4 h at 4C. The tissues were then cryoprotected by being placing them in raising concentrations of sucrose (10-30%) in PBS at 4°C. Tissues were embedded in OCT (RA Lamb Labs) and stored at -80C until required. Twelve micron-thick coronal midbrain sections were mounted onto positive charged slides to perform immunohistochemistry.

### Immunohistochemistry and detection of internalized phage

Prior to fixation, CP cells in culture and CP tissue explants were rinsed in 50 mM Glycine buffer pH 2.8 for 5 min or 10 mM PBS pH 7.4 containing 0.3% Tween-20 (Sigma), respectively. Cells and tissue samples were then rinsed in PBS (3X) and fixed for 20 min at room temperature in 2.2% formaldehyde in 10 mM PBS pH 7.4 containing 2% glucose and 0.02% sodium azide. Cells and explants were rinsed once with PBS, permeabilized in methanol for 6 min, and washed twice again with PBS. Immunofluorescence and immunoperoxidase staining techniques using antibodies to the coat protein M13 phage were used to detect internalized phage in either cultured epithelial cells *in vitro*, CP tissue explants or midbrain coronal brain sections after ICV injection of phage particles. To block non-specific staining, cells and tissue samples were incubated with PBS containing 1% BSA (Jackson Immuno Research), 0.3% Tween 20 (Sigma), and 10% NGS (Jackson Immuno Research) for 20 minutes at room temperature. Excess blocker solution was drained and samples incubated with rabbit anti-bacteriophage (1/200, Sigma) for 1 h at room temperature, rinsed and then incubated with secondary labelled anti rabbit antibodies. For immunofluorescence staining, samples were then incubated for 45 min at room temperature with Alexa^594 ^labelled goat anti-rabbit antibodies (Invitrogen). After washing with PBS, cell and tissue samples were mounted using Vectashield mounting medium containing DAPI (Vector Labs) and visualized under an epifluorescent microscope and confocal microscopy. In some instances peroxidase staining was used to evaluate the distribution of phage particles in the brain after i.c.v. injection. In this instance, after incubation of the tissues with rabbit anti-M13 antibodies, tissue sections were rinsed and incubated with biotinylated goat anti-rabbit IgG (Vector) for 45 min at room temperature. The tissue sections were rinsed in PBS and endogenous peroxidase quenched by incubating the tissue sections in 0.3% hydrogen peroxide (30% H_2_O_2_, Sigma) for 30 min, washed in PBS, and incubated with ABC solution (ABC Kit, Vector Labs) for 30 min. Sections were incubated in Diaminobenzidine (DAB, Vector Labs) for 5-7 min rinsed, dehydrated, cleared in Histoclear and mounted. Immunostaining was visualized under bright field microscopy and DIC optics (Zeiss Axioplan).

### Evaluation of targeting specificity

To investigate whether the internalization of peptide-targeted phage was ligand specific, we performed competition assays using the cognate synthetic peptides displayed on phage. TRCSFB cells or tissue explants were incubated for 2 hr at 37C under 5% CO2 with peptide-targeted phage (10^12 ^particles/ml) 10-15 minutes after having added 200 ng to 1 ug of the cognate synthetic peptide. Peptides were prepared by solid phase synthesis in the University of Birmingham Protein Core Laboratories, evaluated by amino acid analyses and purified to >95% homogeneity.

## Abbreviations

CNS: central nervous system; CSF: cerebrospinal fluid; CP: Choroid plexus; CPe: choroid plexus epithelial; EGF: epidermal growth factor; FCS: fetal calf serum; IGF1: insulin like growth factor 1; i.c.v.: intracerebroventricular; PBS: phosphate buffered saline; NGS: normal goat serum; NHS: normal horse serum

## Competing interests

The authors declare that they have no competing interests.

## Authors' contributions

AMG led the UK team and had primary responsibility for the animal preclinical studies, directing and conducting the rat CNS injuries, the immunohistochemistry contributions to experimental design and helping write the manuscript. WEL assisted in the cell culture, explants, rat CNS and immunohistochemistry experiments. MB led the molecular aspects of the studies including its design, PCR and qPCR, cloning, vectors and plasmids, participating in experimental design and analysis. KS had primary responsibility for staining of sections, the associated QA/QC of cell culture and explant studies, data analyses and figure preparation. TT provided the cell line of immortalized CP cells. EGS supervised and designed experimental approach with explant tissues, guided immunohistochemistry and data analyses. CEJ assisted in generating original hypothesis, interpreting experimental results and manuscript preparation. BPE assisted in design of experiments and manuscript preparation. AB generated the original hypothesis, supervised the experimental designs and data interpretation and wrote the manuscript. All of the authors have read and approve of the final manuscript.
